# New Appliances and Things Medical

**Published:** 1902-03-29

**Authors:** 


### NEW APPLIANCES AND THINGS MEDICAL.

[We shall be glad to receive, at our Office, 28 & 29 Southampton Street Strand, London, W.C., from the manufacturers, specimens of all new preparations and appliances which may be brought out from time to time.]

### SALUS SPITTOON AND CARDBOARD SPITTOON FLASKS.

(Ouventorse Patent. London Agent: George Fingerhut, 7 Mincing Lane, London, E.C.)

These destructible spittoons for phthisical patients are well worthy of the attention of hospital authorities, medical practitioners, and those who are responsible for the care of consumptives. They are made of waterproof cardboard, and can be immediately destroyed by burning after use. These spittoons are made in a variety of shapes, according to the purpose they are intended to subserve. For use in the ward or sick room they are shaped like a deep saucer, with a tongue-like handle, and a lid which fits accurately and securely. For pocket use they are shaped like a cigar case with fixed lid. Sputum may be conveniently received in these spittoons, and carried about the person without danger to others. A little wadding or cotton wool soaked in a convenient antiseptic, and packed at the bottom of these receptacles, would be a wise and simple precaution. Similar vessels may be obtained in a slightly superior quality coated by a special process with white enamel. These receptacles can, if necessary, be soaked in an antiseptic solution, and used over and over again. The cost of these convenient spittoons and vessels is so small that the expense of providing the inmates of hospitals and institutions with them would be altogether insignificant.

